# The Association of Nasal and Blood Eosinophils with Serum IgE Level in Allergic Rhinitis and Asthma: A Case‐Control Study

**DOI:** 10.1002/hsr2.70191

**Published:** 2024-11-07

**Authors:** Mehdi Torabizadeh, Mojtaba Aghaei, Najmaldin Saki, Mohammad A. Vahid, Saeid Bitaraf, Bita Bandar

**Affiliations:** ^1^ Golestan Hospital Clinical Research Development Unit Ahvaz Jundishapur University of Medical Sciences Ahvaz Iran; ^2^ Thalassemia & Hemoglobinopathy Research Center, Health Research Institute Ahvaz Jundishapur University of Medical Sciences Ahvaz Iran; ^3^ Student Research Committee Ahvaz Jundishapur University of Medical Sciences Ahvaz Iran; ^4^ Department of Community Medicine, School of Medicine Ahvaz Jundishapur University of Medical Sciences Ahvaz Iran

**Keywords:** allergic rhinitis, asthma, IgE, nasal eosinophils

## Abstract

**Background and Aims:**

Allergic rhinitis and asthma are two common respiratory diseases with allergic etiology in the world's population. Eosinophils and serum IgE levels have been known as inflammatory allergy markers for many years. This study aimed to evaluate the correlation of nasal and blood eosinophils with serum IgE levels in allergic rhinitis and asthma patients.

**Methods:**

This prospective study was done on patients (*n* = 78) diagnosed with asthma (*n* = 20), allergic rhinitis (*n* = 49), and chronic rhinosinusitis with nasal polyposis (CRSwNP) (*n* = 9) at our hospital in Ahvaz City, Iran. The age of participants in our study ranged from 3 to 73 years, and all of them were subjected to a complete blood count (CBC) test, nasal smear, and determination of serum IgE levels after their consent.

**Results:**

There was no correlation between serum IgE level and nasal eosinophil count (*p* = 0.728) or between serum IgE level and blood eosinophil count (*p* = 0.657); however, a positive correlation was detected between blood and nasal eosinophil levels (*p* = 0.003).

**Conclusion:**

There is no significant relationship between serum IgE level and eosinophil count in the blood and nasal secretions. Serum IgE level and blood or nasal eosinophil count are both useful biomarkers for monitoring allergic rhinitis and asthma individually, but no diagnostic conclusion can be drawn from their correlation.

## Summary

- There was no correlation between serum IgE level and nasal eosinophil count.
- No correlation was observed between serum IgE level and blood eosinophil count.
- An elevated nasal eosinophil count is correlated with an elevated blood eosinophil level.

**Abbreviations**
AR: allergic rhinitis
ARIA: Allergic Rhinitis and its Impact on Asthma
CBC: complete blood cell count
CRSwNP: chronic rhinosinusitis with nasal polyposis
EDTA: ethylene diamine tetra acetic acid
ELISA: enzyme‐linked immunosorbent assay
IgE: Immunoglobulin E
WHO: world health organization

## 1 Introduction

According to an estimate by the World Health Organization (WHO) in 2019, 262,000,000 people in the world are afflicted with asthma. Asthma occurs more often in those who have an allergic background such as allergic rhinitis (AR) [1, 2]. In addition, the World Allergy Organization estimates that 10%–30% of the world's population suffers from allergic rhinitis [3]. The study of relationship between asthma and AR has been reviewed under the title “Allergic Rhinitis and its Impact on Asthma” (ARIA) program in WHO.

Studies have shown that AR increases the risk of asthma up to threefold. Having AR diminishes the quality of treatment in asthma patients. The pathophysiology of these two diseases has many similarities, including the type of inflammation and the secreted mediators. In AR, the immediate response to allergens leads to the release of histamines, leukotrienes, and prostaglandins from mast cells, leading to symptoms such as nasal congestion and runny nose due to increased permeability of vessels in the upper respiratory tract [4, 5]. In asthma, these mediators cause bronchospasm in the lower respiratory system [6].

As an inflammatory marker of allergy, the level of eosinophils in nasal secretions is elevated in patients with airway inflammation. Studies have shown that eosinophils in nasal secretions increase in patients with asthma, as well as in allergic and nonallergic rhinitis [7, 8]. In general, allergic patients have higher serum Immunoglobulin E (IgE) levels compared to nonallergic individuals. Serum IgE level can be used as an indicator of allergic disease. However, it is important to note that not all patients with elevated IgE levels have clinically significant allergies [9, 10]. Therefore, the diagnosis of allergy should be based on a combination of clinical history, physical examination, and laboratory testing, including measurement of IgE levels and skin testing [11, 12]. Based on statistical studies conducted in Iran, the average prevalence of asthma in the country is 10% and there is a high frequency of asthma and allergy among all age groups in Ahvaz city of Khuzestan province [13, 14, 15, 16, 17]. Therefore, considering the disease prevalence in this region and due to the genetic differences among various races and populations, it is of high importance to study this category of patients in this region. The purpose of this pilot research was to investigate the correlation between serum IgE levels and the amount of nasal and blood eosinophils in patients suffering from allergic rhinitis and asthma in southwestern Iranian patients.

## 2 Materials and Methods

### 2.1 Sample Collection

We studied 78 patients referred to Center of Asthma and Allergy of Golestan Hospital in Ahvaz city of Iran. The patients were diagnosed into three groups of asthma, allergic rhinitis and chronic rhinosinusitis with nasal polyposis (CRSwNP) by an allergy and immunology specialist. Nasal smear samples were taken from nostrils of all patients using a cotton swab, and their blood was collected in a tube containing ethylene diamine tetra acetate (EDTA) to perform CBC test. Besides, patients' sera were used to evaluate IgE levels.

### 2.2 Eligibility Criteria

Considering the prevalence of respiratory allergies in southwest Iran, the patients all had airborne allergies diagnosed with asthma, allergic rhinitis ‐both seasonal and perennial types were included‐ and CRSwNP. We included the on‐treatment patients consecutively without an age limit, sensitized to more than one airborne allergen (polysensitized) based on the prick skin test. Patients with skin or food allergies were excluded.

### 2.3 Test Methods

**Figure 1:**
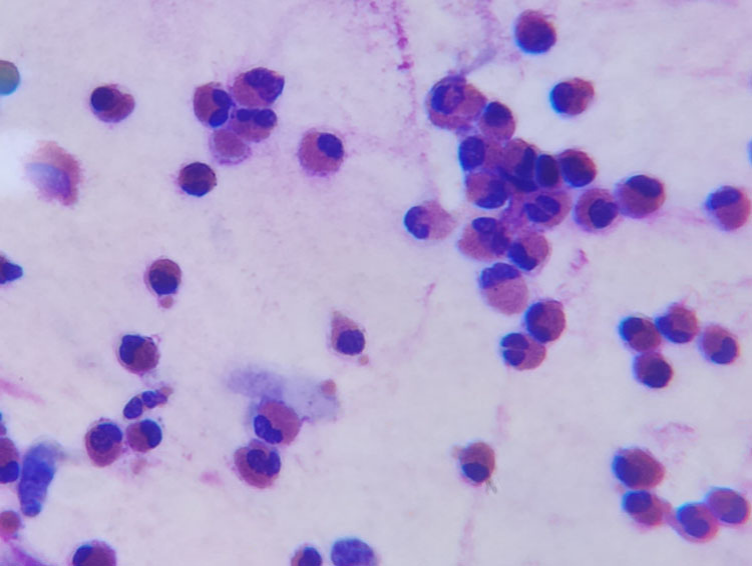
Infiltration of eosinophils in nasal secretion smear of a patient diagnosed with allergic rhinitis. (Wright–Giemsa staining, magnification 40x).

From each patient, two or three nasal smear slides were prepared, which were stained with Wright‐Giemsa after drying. Then, the nasal smear was examined with an optical microscope, and cell differentiation was performed to assess the percentage of eosinophils, neutrophils, basophils/mast cells and goblet cells (Figure 1). Serum IgE levels were measured using enzyme‐linked immunosorbent assay (ELISA) technique according to the instructions of the manufacturer. The serum IgE levels were measured by Mononind lnc. IgE ELISA kits and the absorbance was measured at 450 nm using an ELISA plate reader. The normal range for serum IgE levels due to the kit's instructions is considered up to 46 IU/mL in infants, up to 280 IU/mL in children, and up to 200 IU/mL in adults.

### 2.4 Statistical Analysis

To analyze the data, we used descriptive statistics methods, including frequency distribution tables, graphs, and indices of central tendency, and appropriate dispersion was used to describe the studied variables. Then, the normality of the distribution of quantitative variables was checked with the Kolmogorov–Smirnov test. The chi‐square test and Pearson correlation coefficient were used to study the correlation between qualitative variables, and an independent *t*‐test or its non‐parametric equivalent was used to compare quantitative values between the two groups. The scatter plot was used to show the correlation visually. The significance level of the tests is considered to be smaller than 0.05. Data analysis was done using STATA 12 software.

## 3 Results

In total, 78 participants were recruited to our study. We further classified the participants into three groups: asthma (*n* = 20), allergic rhinitis (*n* = 49), and chronic rhinosinusitis with nasal polyposis (CRSwNP) (*n* = 9). Baseline characteristics of participants are presented in Table 1. There were 27 males and 22 females in allergic rhinitis group with mean age of 24.08, while asthma group consisted of 12 males and eight females with mean age of 31.9, and chronic rhinosinusitis group included six males and three females with mean age of 27.11. There was no significant difference in terms of gender or age between these groups.

**Table 1:** Demographic characteristics of lab tests in the participants.

		Allergic rhinitis	Asthma	CRSwNP	
		*N* = 49 Mean ± SD	*N* = 20 Mean ± SD	*N* = 9 Mean ± SD	*p*‐value
Age		24.08 (18.60)	31.90 (19.47)	27.11 (16.76)	0.29
Sex	Female	22 (45%)	8 (40%)	3 (33%)	0.84
	Male	27 (55%)	12 (60%)	6 (67%)	
Nasal Eosinophils%		8.89 (21.29)	6.69 (14.59)	9.11 (17.92)	0.91
Nasal Basophils%		0.20 (0.73)	4 (17.89)	0.33 (1)	0.28
Nasal Neutrophils%		40.39 (39.66)	41.10 (42.05)	43.22 (40.01)	0.98
Nasal Goblet cells%		50.51 (41.79)	44.20 (39.90)	47.33 (43.18)	0.85
Total serum IgE level (IU/ml)		341.32(511.94)	363.19 (338.89)	828.51 (1517.89)	0.13
Blood WBC count (10^3/µL)		8.71 (3.02)	9.9 (2.63)	9.04 (1.76)	0.29
Neutrophil%		51.04 (11.39)	53.45 (16.06)	58.11 (12.64)	0.30
Lymphocyte%		38.19 (11.65)	34.85 (14.26)	32.56 (9.93)	0.33
Monocyte%		6.64 (2.27)	6.4 (2.26)	5.33 (1.80)	0.27
Eosinophil%		3.16 (3.54)	4.2 (3.56)	3.11 (2.67)	0.51
Basophil%		0.82 (0.42)	0.9 (0.45)	0.78 (0.44)	0.72

Abbreviations: CRSwNP, chronic rhinosinusitis with nasal polyposis; IgE, Immunoglobulin E; WBC, white blood cell.

The percentage of nasal eosinophils, nasal basophils, nasal neutrophils, and nasal goblet cells did not show a significant difference between the three groups (all *p* > 0.05). Furthermore, these three groups were not significantly different in terms of total serum IgE level (*p* = 0.13).

### 3.1 Correlation Between Total Serum IgE Level and Eosinophils

**Figure 2:**
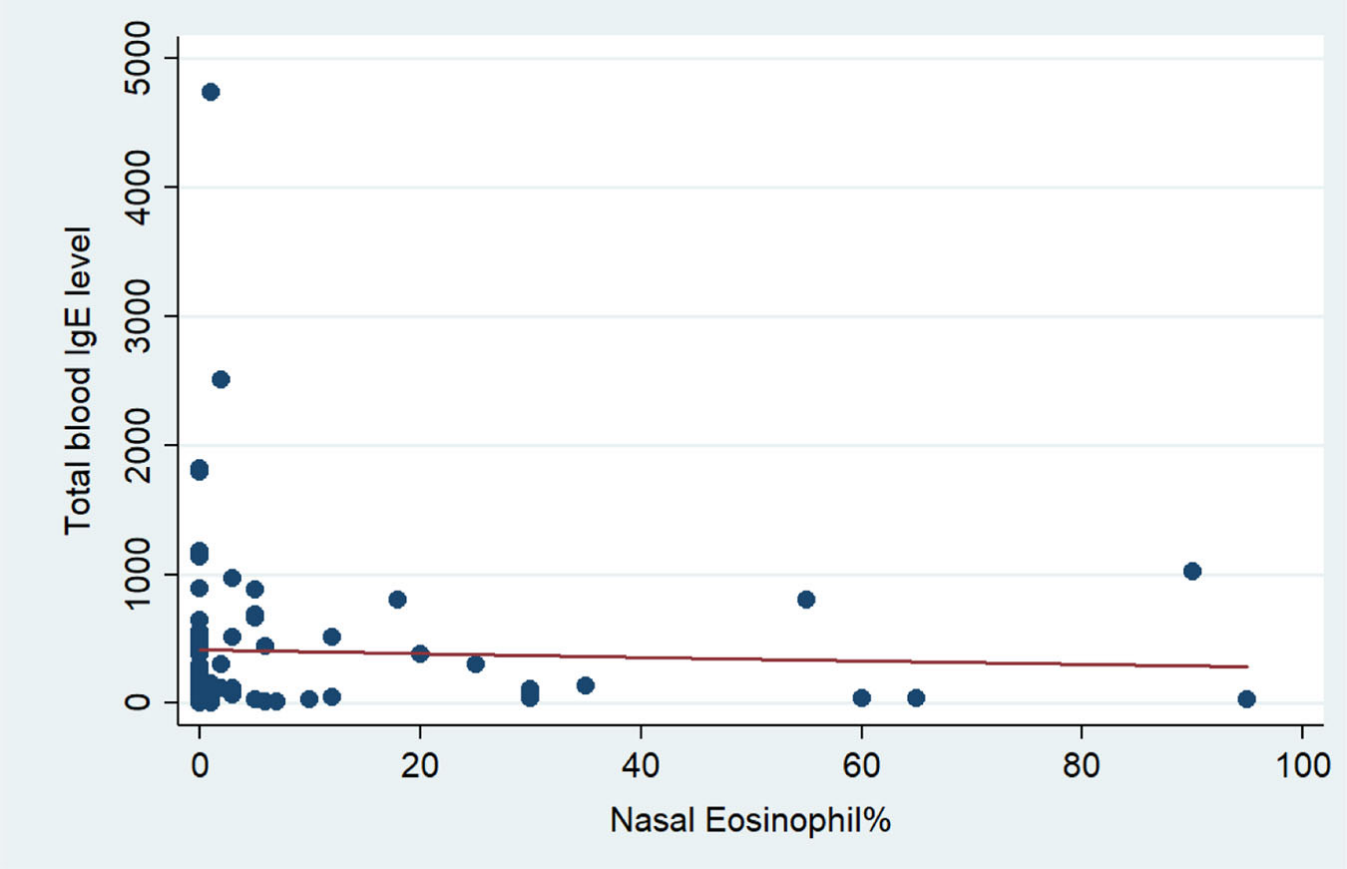
Correlation between serum IgE level and nasal Eosinophil.

**Figure 3:**
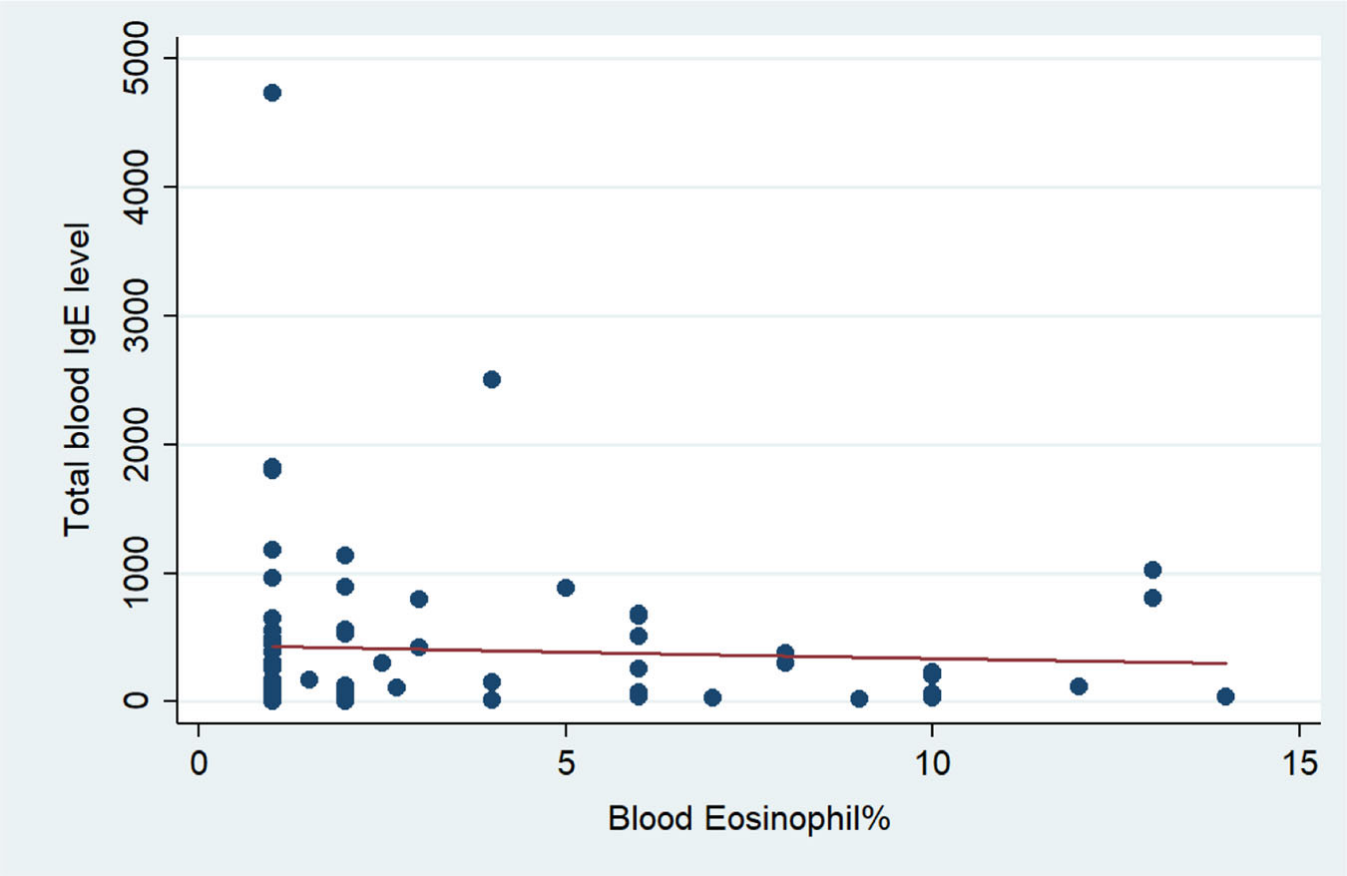
Correlation between serum IgE level and blood Eosinophil.

Regardless of disease type, there was no correlation between total serum IgE levels with nasal eosinophils (*p* = 0.728) (Figure 2 and Table 2), or with blood eosinophils (*p* = 0.657) (Figure 3 and Table 2). Furthermore, there was no significant correlation between total serum IgE levels and nasal or blood eosinophils in patients with allergic rhinitis, asthma, or CRSwNP (*p* > 0.05) (Table 4).

**Table 2:** Correlation between serum IgE level and blood and nasal Eosinophils.

Variables	IgE
Nasal eosinophil	−0.040
	(0.728)
Blood eosinophil	−0.051
	(0.657)

### 3.2 Correlation Between Nasal and Blood Eosinophil

There was a positive correlation between blood and nasal eosinophil levels (*p* = 0.003), indicating that an elevation in blood eosinophils is correlated with an increase in nasal eosinophils (Table 3). This observation was a function of pooling data from all three groups irrespective of disease type.

**Table 3:** Correlation between nasal and blood eosinophil.

Variables	Nasal eosinophil
Blood eosinophil	correlation coefficient = 0.332
	*p* value = 0.003

## 4 Discussion

### 4.1 Overview

This study aimed to explore the correlation between nasal and blood eosinophils with serum IgE levels in patients diagnosed with allergic rhinitis, asthma, and CRSwNP. A total of 78 participants were categorized into these three groups. Our analysis showed no significant differences in nasal eosinophils, basophils, neutrophils, goblet cells, or total serum IgE levels among these groups. Additionally, we found no significant correlation between serum IgE levels and eosinophil counts, either in blood or nasal secretions, across the patient groups. However, a significant positive correlation was identified between nasal and blood eosinophil levels, suggesting a potential link between systemic and localized eosinophilic inflammation.

### 4.2 Interpretation of the Main Findings

The main findings of this study demonstrate that serum IgE levels do not correlate with eosinophil counts in either blood or nasal secretions in patients with allergic rhinitis, asthma, or CRSwNP. This result is consistent with some studies, such as that of Gerday et al., which found that elevated IgE levels do not always align with increased eosinophil counts in sputum or blood, indicating that serum IgE and eosinophilic inflammation may not be directly related in all allergic conditions (*p*‐value > 0.05) (Table 4).

**Table 4:** The association of nasal and blood eosinophils with serum IgE Level in allergic rhinitis and asthma.

	Allergic rhinitis	Asthma	Chronic rhinosinusitis
IgE & blood Eosinophil correlation	0.008	0.062	−0.315
*p*‐value	0.958	0.796	0.408
IgE & nasal Eosinophil correlation	−0.019	−0.215	−0.057
*p*‐value	0.898	0.363	0.883

This contrasts with other studies that have reported a significant association between IgE levels and eosinophilia in similar populations, highlighting the complexity and variability of allergic responses (Table 5). Importantly, the observed positive correlation between nasal and blood eosinophils across all patient groups suggests that blood eosinophilia may reflect eosinophilic activity in nasal tissues, which could be indicative of a broader systemic allergic response.

**Table 5:** Some latest articles on the association of IgE levels and Eosinophils.

Study	Results	Reference
Gerday et al.	The majority of asthmatics had a raised total serum IgE and eosinophilic phenotype atopy.	[16]
Badar et al.	The study showed a significant positive relationship between baseline FeNO and peripheral blood eosinophils and total IgE.	[17]
Gomes de Amorim et al.	The study showed that vitamin D level < 24 ng/mL has a positive correlation with IgE levels and eosinophil counts in asthmatic children.	[18]
Kumar et al.	There was a significant association between peripheral eosinophil count, sputum eosinophil count, and elevated serum IgE with severe persistent asthma. Sputum eosinophilia can be a biomarker for severe persistent asthma.	[19]

### 4.3 Investigation of the Relationship Between Total Serum IgE Level and Eosinophils

Our findings revealed no significant correlation between total serum IgE levels and eosinophil counts in both nasal secretions and peripheral blood, either when considering the entire cohort or within individual disease groups. This lack of association suggests that while serum IgE is a key player in allergic reactions, its levels may not reliably indicate the extent of eosinophilic inflammation in allergic rhinitis, asthma, or CRSwNP. The broad age range of our study participants (3–73 years) could have contributed to this variability, as immune responses, including IgE production and eosinophilia, can vary significantly with age. On the other hand, the pathogenesis of respiratory allergies is the same in all ages, but with increasing age, it may become more difficult to control and treat the disease due to the possibility of worsening symptoms. Therefore, the disease mechanism is similar at all ages. Of course, infant and senile patients were excluded from the study due to their compromised immune systems. Additionally, all patients had polysensitized airborne allergy during whom on‐treatment phase but the duration of the disease was not accounted for in our analysis, which could also be a confounding factor.

### 4.4 Investigation of the Correlation Between Nasal and Blood Eosinophils

The significant positive correlation observed between nasal and blood eosinophils indicates that increased eosinophil counts in blood are associated with increased eosinophil levels in nasal secretions, regardless of the underlying allergic condition. This correlation suggests a potential systemic aspect of eosinophilic inflammation that is consistent across different allergic diseases. Our findings align with the concept that blood eosinophils can migrate into nasal tissues during allergic reactions, reflecting a coordinated response of eosinophils between the bloodstream and local tissues.

### 4.5 Biological Mechanisms Underlying These Connections

The biological mechanisms underlying the observed relationships likely involve the common pathways of eosinophil recruitment and activation in allergic responses. Allergic reactions typically involve IgE‐mediated activation of immune cells such as mast cells and basophils, leading to the release of cytokines like IL‐5 and eotaxins, which are pivotal in the recruitment and activation of eosinophils [20, 21, 22]. Despite these shared pathways, our study found no direct link between serum IgE levels and eosinophil counts, suggesting that eosinophilic activity in blood and nasal tissues may be regulated by additional factors, such as local tissue signals or variations in immune responses due to age or genetic predispositions [23, 24, 25]. The significant correlation between blood and nasal eosinophils points to the possibility of systemic inflammation that promotes eosinophil infiltration into nasal tissues during allergic responses, independent of serum IgE levels. According to previous studies, there might be a relationship between nasal provocation test and localized nasal IgE levels [10]. Therefore, future studies are recommended to investigate the relationship between nasal eosinophils and localized nasal IgE levels.

However can somehow be associated with other local responses in the nose and can also be a suggestion to be included in the continuation of the Authors research regarding upper airway allergy, to which they are encouraged.

### 4.6 Strengths and Limitations

A notable strength of this study is the inclusion of multiple allergic conditions, which allows for a broader understanding of the relationship between serum IgE levels and eosinophilia across different allergic diseases. The use of both nasal and blood samples provides valuable insights into the systemic and localized nature of eosinophilic inflammation. However, several limitations must be acknowledged. For instance, the duration of the disease was not evaluated in this study, despite being an important factor that could influence both IgE levels and eosinophilia. In addition, our study did not explicitly address whether patients with overlapping phenotypes—those presenting symptoms of multiple allergic conditions simultaneously—were included or excluded, which could have influenced the results. Understanding whether such overlaps were present is crucial, as they could alter the interpretation of eosinophil correlations across different conditions.

These limitations highlight the importance of careful interpretation and suggest avenues for future research, such as including a more clearly defined selection method, and considering the age range and duration of disease. Future studies should aim to address these aspects to better understand the complex interplay between IgE levels, eosinophil counts, and allergic diseases.

## 5 Conclusion

No significant correlation exists between serum IgE levels and blood or nasal eosinophil counts in allergic rhinitis and asthma. Serum IgE levels and blood or nasal eosinophil counts can be used individually for patient follow‐up because of their diagnostic role in allergic rhinitis and asthma, but no significant relationship was detected between them. Nevertheless, an increased blood eosinophil level is correlated with elevated nasal eosinophil levels.

## Author Contributions

**Mehdi Torabizadeh:** writing–original draft; conceptualization. **Mojtaba Aghaei:** writing–original draft. **Najmaldin Saki:** writing–original draft. **Mohammad A. Vahid:** methodology; writing–original draft. **Saeid Bitaraf:** formal analysis. **Bita Bandar:** writing–original draft; writing–review and editing; supervision.

## Ethics Statement

All the procedures performed in this study involving human participants were in accordance with ethical standards of local Ethics Committee of Ahvaz Jundishapur University of Medical Sciences (AJUMS) (IR. AJUMS. REC.1402.376) as well as 1964 Helsinki declaration.

## Conflicts of Interest

The authors declare no conflicts of interest.

## Transparency Statement

The lead author Bita Bandar affirms that this manuscript is an honest, accurate, and transparent account of the study being reported; that no important aspects of the study have been omitted; and that any discrepancies from the study as planned (and, if relevant, registered) have been explained.

## Data Availability

All data generated or analyzed during this study are included in this article. The corresponding author can be contacted for more information in this regard.
